# Endometrial carcinosarcoma: a poor prognosis debut with favourable therapeutic outcome

**DOI:** 10.3332/ecancer.2022.1472

**Published:** 2022-11-17

**Authors:** María Fernanda Toro-Wills, Angelina Álvarez-Londoño, Abraham Hernández-Blanquisett, Fernando Salas Marquez, María Cristina Martínez-Ávila

**Affiliations:** 1 Department of Gynecology, Centro Hospitalario Serena del Mar, Cartagena 130001, Colombia; 2 Department of Radiation Oncology, Cancer Institute, Centro Hospitalario Serena del Mar, Cartagena 130001, Colombia; 3 Department of Clinical Oncology, Cancer Institute, Centro Hospitalario Serena del Mar, Cartagena 130001, Colombia; 4 Department of Gynecology Oncology, Centro Hospitalario Serena del Mar, Cartagena 130001, Colombia

**Keywords:** endometrial cancer, carcinosarcoma, therapeutic approach, chemotherapy, radiotherapy, overall survival

## Abstract

Endometrial carcinosarcoma (ECS) is a rare, highly aggressive disease characterised by a biphasic growth of malignant epithelial (carcinomatous) and mesenchymal (sarcomatous) components. Clinically, it cannot be distinguished from endometrial carcinoma or uterine sarcoma. The definitive diagnosis can only be made based on histological examination and immunohistochemistry. To date, there aren’t standardised treatment protocols for its management. We report a case of a 73-year-old patient who presented postmenopausal abnormal uterine bleeding and was diagnosed with ECS. A non-conventional treatment approach was conducted with favourable oncological outcomes.

## Introduction

Endometrial carcinosarcoma (ECS), also known as malignant mixed Müllerian tumour, is a rare, highly aggressive disease [1]. It accounts for approximately 2%–5% of all uterine neoplasms, causing around 16% of all deaths and an estimated 5-year overall survival (OS) of 33%–39% [2].

ECS are characterised by a biphasic growth of malignant epithelial (carcinomatous) and mesenchymal (sarcomatous) components which could be of diverse histological origin: homologous (endometrial stromal sarcoma, fibrosarcoma, leiomyosarcoma) or heterologous (osseous, cartilaginous, and rhabdomyoblastic) cells [3]. Clinically, ECS cannot be distinguished from endometrial carcinoma or uterine sarcoma. The definitive diagnosis can only be made based on histological and immunohistochemistry examination [4].

Owing to its microscopic diversity and low frequency of cases, standardised treatment protocols are not available [5, 6]. Proposed treatment includes cytoreduction surgery for medically operable patients and adjuvant therapy that might be radiotherapy or systemic therapy based on surgical and histopathological findings [4, 6].

Herein, we present the case of a 73-year-old patient who presented painless abnormal uterine bleeding for 1 year and was diagnosed as a IIIB endometrial neoplasm with evidence of parametrial infiltration not candidate for surgical management. A non-conventional treatment with concomitant chemo-radiotherapy was done with favourable oncological results which allowed an interval surgical procedure with complete debulking.

## Case report

A 73-year-old patient consulted for having 1 year of painless vaginal bleeding. She was initially evaluated by gynaecologists who document endometrial thickening and began a diagnostic approach.

Regarding her background history, the patient had arterial hypertension since 2005. In 2021, she had an angioplasty + medicated stent because of a severe coronary artery disease of one vessel. Her prescribed medications consisted of metoprolol 100 mg a day, ticagrelor 90 mg a day and acetylsalicylic acid 100 mg a day. She was allergic to penicillin and denies alcohol or tobacco use. OB/GYN history g2p2, with two caesarean sections.

Physical examination did not reveal masses or megaly. The inguinal region was without evidence of lymphadenopathy. In the gynaecological examination, a large tumour lesion of approximately 12 cm, which occupies the entire vagina was documented. The anterior vaginal wall had a nodular implant of approximately 1 cm. Uterus and annexes were non-delimited and there was bilateral parametrial tumoral involvement.

She has the following imaging results: resonance of abdomen and pelvis with enlarged uterus 88 × 84 × 94 mm. 12 mm thickened endometrium. At the left adnexal level, a cystic lesion of 13 mm, there was no free fluid in the cavity.

An endometrial biopsy was performed revealing an ECS. Complementing the histopathological diagnosis with imaging studies and physical examination, it is staged as a IIIB endometrial tumour with evidence of parametrial infiltration. She was referred for an oncological gynaecologist evaluation, and he considered the patient was not a candidate for primary surgical management and was derived to our cancer institute for a palliative care intention management.

**Figure 1.**
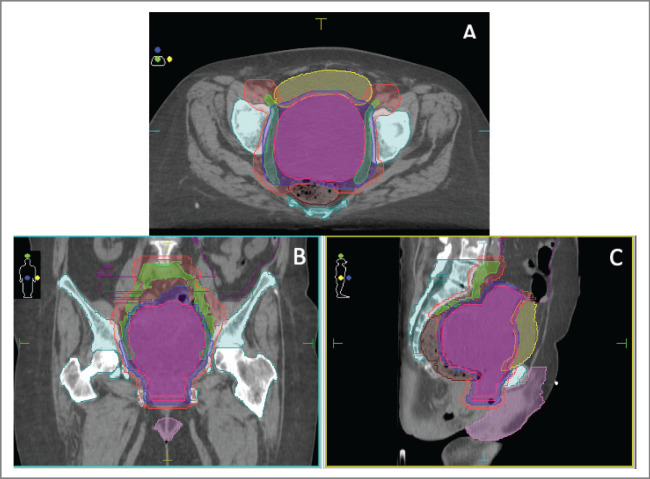
(a–c): Treatment plan in axial (a), coronal (b) and sagittal (c) views with VMAT technique.

She was admitted to our institution where a multidisciplinary board decided that the patient had a good performance status (PS). PS scores measured the patient’s ability to perform certain activities of daily living without the help of others. We calculated an Eastern Cooperative Oncologic Group of 1 describing she was symptomatic but an ambulatory nature patient, and a Karnofsky Performance Scale Index of 90 revealing she was capable of normal activity with few symptoms or signs of disease. A non-conventional approach with concomitant chemoradiotherapy (cisplatin 40 mg/day, unique cycle, 6 weeks: days 1, 8, 15, 22, 29, 36) and volumetric modulated arc therapy (VMAT) technique external radiotherapy, total dose of 54 Gy in fractions of 1.8 Gy, a sequential schedule planning target volume 1 (PTV1) (45 Gy) and a boost on PTV11 (9 Gy) was proposed (Figure 1).

**Figure 2.**
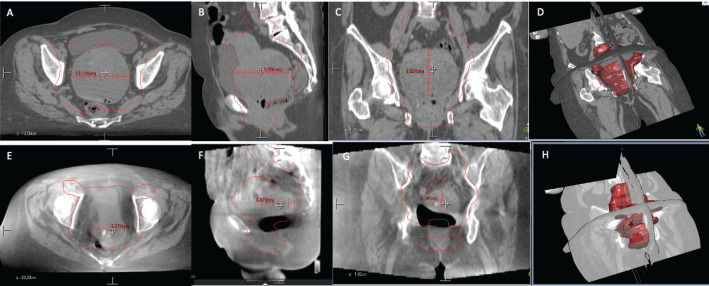
Comparison of CT images. (a–d): (Upper row, a–d) Before radiotherapy, uterus measures were 85.2 × 95.8 x 10.4 mm. (e–h): (Lower row, e–h) After radiotherapy, uterus measures were 43.4 x 60.7 x 62.7 mm.

After submitting the patient to concomitant chemo-radiotherapy, a favourable clinical response was achieved, bleeding ceased and control images showed a reduction of approximately 60% of the tumour (Figure 2). She was reassessed by oncological gynaecologist and was programmed for an interval surgery.

**Figure 3.**
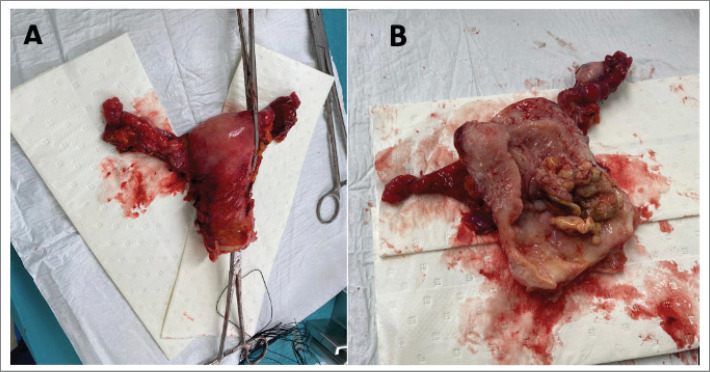
Macroscopic view of the sample (uterus + adnexa). (a): Uterus, fallopian tubes and ovaries after surgical procedure. (b): Longitudinal incision of the sample with evidence of tumoral mass coming from posterior uterine wall.

She was scheduled for an extended total abdominal hysterectomy (extrafascial type b1) + bilateral salpingo oophorectomy + total omentectomy. Intraoperatively, a uterus +/− 6 cm in size with evidence of a 2 × 3 cm fundic and posterior myoma was documented. The surgical specimen was dissected with evidence of a macroscopically healthy cervix, an irregular neoplastic mass of approximately 6 × 3 cm depending on the posterior wall of the uterus. Bilateral macroscopically normal adnexa. Macroscopically normal omentum. No para-aortic, pelvic or retroperitoneal lymphadenopathies, as well as intra-abdominal lymphadenopathy were found. Douglas cul-de-sac, parametrial, uterus sacral ligaments and rectovaginal septum were free from gross lesion (Figure 3).

A histopathologic diagnosis of a high-risk ECS with immunohistochemistry confirmation that showed epithelial component and displayed heterologous stromal elements. Immunoperoxidase markers were made with Cytokeratin 7 (CK7), Paired-box gene 8 (PAX8), oestrogens receptors, smooth muscle actin, Cluster of differentiation 10 (CD10), P53 and Ki67. Tumour cells showed strong reactivity with a membrane pattern for CK7, a nuclear pattern for PAX8 and a focal pattern for oestrogens receptors. P53 immunostaining showed mutation-type and a diffuse over-expression with a nuclear pattern. A Ki67 cell proliferation index of 90%.

In post-operative consultations, patient continued asymptomatic and free of disease for 6 months. Unfortunately, a few months later, she died due to non-oncological related causes.

## Discussion

ECS, due its endometrial origin, is staged as an endometrial carcinoma/cancer (EC) rather than a uterine sarcoma [7, 8]. The International Federation of Gynecology and Obstetrics defined the staging system from I to IV according to the extension of the disease [8]. The stage is the most important factor in choosing treatment [8]. For EC stages I-II, surgery is the first treatment, depending on the histopathology classification adjuvant therapies: radiation therapy, chemo, or both may be given [8].

On the other hand, as stages III and IV are tumours extending beyond the uterus, usually they are not candidates for surgery as primary treatment; chemo and radiotherapy, as well as hormone or biological therapy are the management strategies with palliative care intentions (reduce pelvic pain or bleeding) [7]. Even though, in some cases, they can be taken into surgery if debulking is possible and require adjuvant therapy [7].

Despite the fact our patient had an advanced stage ECS, she had good PS; this was crucial for the multidisciplinary board decision to proceed with a neoadjuvant chemoradiation obtaining promising results. The response to the proposed treatment was a significant shrinkage of the carcinosarcoma from 85.2 × 95.8 × 10.4 mm to 43.4 x 60.7 x 62.7 mm generating the opportunity of a satisfactory cytoreduction surgery. A surgery that was never on the table after the initial diagnosis. Follow-up scans demonstrated a patient in remission, free of disease after a non-standard management of ECS.

Due its divergent behaviour and prognosis, the recommendations for managing ECS are scarce, it has been based on expert opinions, small retrospective studies or non-randomised trials potentially biased by unmeasured confounders. There’s no consensus in management or standard of care for ECS [5].

Several studies confirm the clear benefit of chemotherapy for ECS on OS in this patient population [9–11]. Radiotherapy’s role, doses and type are still a controversial topic that is under evaluation [5, 6]. The radiation prescription dose in endometrial cancer is commonly 1.8–2.0 Gy daily fractions up to 36–68 Gy (median 54 Gy) [6]. Our patient received VMAT which is a novel radiation therapy technique that delivers the radiation dose continuously as the treatment machine rotates [12]. VMAT allows a reduction dose to organs at risk (bladder, bowel and rectum), with improved planning target volume (PTV) coverage; hence it increases dose homogeneity in the target volume and decreases the treatment delivery time [12]. The benefit of our radiotherapy approach guided by sequencing tomography imaging in each session gave us the opportunity of a novel surgical approach in a non-surgical initial scenario.

The evidence in the use of systemic therapy in advanced stage ECS is a prospective study area. In 2015, a National Cancer Database analysis evaluated the rates of chemo-radiotherapy use as primary treatment in a significant cohort of patients (10,609) with uterine carcinosarcoma with different stages of the disease [13]. The study showed adequate tumour response after the use of chemo-radiation in advanced stages improving the OS (65 months (95% CI: 56–77)) [13]. Our patient’s outcome showed that alternative treatment could impact in OS rates despite initial prognosis.

## Conclusions

ECS is a rare entity with an aggressive histopathological presentation and limited OS. The mesenchymal component may contain cell types native to the uterus, and resemble high-grade undifferentiated sarcoma or fibrosarcoma; or extrauterine subtypes that frequently appear to be rhabdomyosarcomas or chondrosarcomas; this classification provides stratification and guides management. A non-traditional approach proceeding with chemo-radiation therapy allowed a satisfactory cytoreduction surgery with disease-free survival. This novel approach showed promising results and should be contemplated among the management possibilities of patients with advanced ECS and good PS. Anyway, further studies are required to standardise this treatment and improve outcomes.

## Conflicts of interest

The authors declare that there are no conflicts of interest.

## Funding

None.
